# Supplementary material for: Dynamic BMP signaling polarized by Toll patterns the dorsoventral axis in a hemimetabolous insect
Source: eLife. 2015 May 12;4:e05502. doi: 10.7554/eLife.05502 (PMC4423117; doi:10.7554/eLife.05502)
Supplement: Supplementary file 1. — PCR primers for production of ISH probes and dsRNA. DOI: http://dx.doi.org/10.7554/eLife.05502.024 [file elife05502s001.docx]

|  | ***Gene*** | **Primer** | **Sequence (5'-3')** | **Product size (bp)** |
| --- | --- | --- | --- | --- |
| **Probes** | *twist (twi)* | F | TCGGAGCTTTGCCGAGAC | 630 |
|  |  | R | GGAATAGTAACATTTCTGGCTTGG |  |
|  | *single-minded (sim)* | F | CCTCCATCATCAGACTGACC | 684 |
|  |  | R | GCGAGAAGTGTATGATGAGAATATC |  |
|  | *short gastrulation (sog)* | F | GTTTGGCAAGTCGTTTCGTA | 1182 |
|  |  | R | TTCTACCAGCCTTGGTGAGG |  |
|  | *muscle segment homeobox (msh)* | F | ACCTGCGGAAGCACAAGC | 1032 |
|  |  | R | TCTGATTTGCTATAATAATGAACTGC |  |
| **dsRNA** | *decapentaplegic (dpp)* | F | ACGCGGGGAGTAGCTAGCC | 329 |
|  |  | R | TTGCGGACCTCACATGGCGT |  |
|  | *decapentaplegic (dpp)* | F | TCCTGGTGGAAGTGGCGAG | 486 |
|  |  | R | TTCCTACATCCACAGCCAACC |  |
|  | *short gastrulation (sog)* | F | GTTTGGCAAGTCGTTTCGTA | 1182 |
|  |  | R | TTCTACCAGCCTTGGTGAGG |  |
|  | *short gastrulation (sog)* | F | ATTGCAGTTGACCCACAATG | 990 |
|  |  | R | CCGTCGATTACACTGAGCAA |  |
|  | *twisted gastrulation (tsg)* | F | AAGTGATTCTCTGAAGGA AGTG | 555 |
|  |  | R | TTGGCTGGATCAACTTCTTGT |  |
|  | *twisted gastrulation (tsg)* | F | ACAAGAAGTTGATCCAGCCAA | 772 |
|  |  | R | TTACTGGATGAACATATGTCT |  |
|  | *tolloid (tld)* | F | CTTGGTTCCTATGTCTGCTGGTGTC | 197 |
|  |  | R | AGTCTTATTCGGTGGCCTGGAGTAG |  |
|  | *Toll (Toll1)* | F | AGATATGGCTATACTCTCACCGATT | 479 |
|  |  | R | GAGGGAGAGCATAGCGGAGT |  |
|  | *dorsal1 (dl1)* | F | CAGACAATGTGACAAAGCCATA | 557 |
|  |  | R | TGAGAAGATCAAGTGTGGCTTT |  |

**Supplementary File 1: PCR primers for production of ISH probes and dsRNA**
